# Stability of Daptomycin in Dextrose and Icodextrin-Based Peritoneal Dialysis Solutions

**DOI:** 10.1155/cjid/5553355

**Published:** 2025-03-27

**Authors:** Kai Ming Chow, Siu Kwan Wo, Simon Wai Yin So, Phyllis Mei Shan Cheng, Keary Rui Zhou, Wai Li Lim, Joan Zhong Zuo, Philip Kam Tao Li

**Affiliations:** ^1^Department of Medicine and Therapeutics, Prince of Wales Hospital, The Chinese University of Hong Kong, Ma Liu Shui, Hong Kong; ^2^Carol & Richard Yu Peritoneal Dialysis Research Centre, The Chinese University of Hong Kong, Ma Liu Shui, Hong Kong; ^3^School of Pharmacy, Faculty of Medicine, The Chinese University of Hong Kong, Ma Liu Shui, Hong Kong; ^4^Pharmacy Department, Alice Ho Miu Ling Nethersole Hospital, Tai Po, Hong Kong; ^5^Pharmacy Department, Prince of Wales Hospital, Shatin, Hong Kong

## Abstract

**Background:** With emerging antibiotic resistance, many patients on peritoneal dialysis require newer antibiotic treatment such as daptomycin. Inadequate clinical information exists across different peritoneal dialysis solutions, including icodextrin, for the stability of intraperitoneal daptomycin. To guide the clinical practice of intraperitoneal daptomycin treatment, we need to establish the stability of daptomycin at dextrose concentration higher than 1.5% and icodextrin, as well as the duration of stability.

**Methods:** We tested the stability of daptomycin in three types of peritoneal dialysis bags (UltraBag dextrose 2.5%, UltraBag icodextrin 7.5%, and Stay-Safe Balance 2.3%). Daptomycin was reconstituted with water for injection (50 mg/mL), followed by administration to peritoneal dialysis bags to obtain the final daptomycin concentrations of 70 μg/mL (equivalent to 140 mg/2L, the maintenance level) and 245 μg/mL (equivalent to 490 mg/2L, the loading level). The bags were then placed at ambient temperature (25°C) followed by withdrawing 5 mL samples at 0, 4, 8, 12, 24, and 48 h for UltraBag dextrose 2.5% and UltraBag icodextrin 7.5% and 0, 4, 8, 12, and 24 h for Stay-Safe Balance 2.3%. The concentrations of daptomycin in the collected samples were quantified by high-performance liquid chromatography with diode array detector (HPLC-DAD).

**Results:** Under ambient condition, daptomycin was stable at maintenance level in UltraBag dextrose 2.5% for 48 h and in UltraBag icodextrin 7.5% or Stay-Safe Balance 2.3% for 24 h. For loading level, daptomycin was stable in UltraBag dextrose 2.5% and Stay-Safe Balance 2.3% for 12 h and in UltraBag icodextrin 7.5% for 48 h.

**Conclusions:** Current stability results support and guide the use of intraperitoneal daptomycin in different dialysis solutions. Patients with peritonitis requiring icodextrin exchange and assisted preparation of daptomycin can benefit from nurses who provide daily home visit based on our stability results.

## 1. Introduction

The mainstay of peritoneal dialysis–related peritonitis treatment is intraperitoneal antibiotics [1, 2]. With emerging antibiotic resistance, there is unmet need of testing stability and compatibility of various classes of antibiotics in dextrose-based and icodextrin-based peritoneal dialysis solutions. As highlighted by the recent International Society for Peritoneal Dialysis (ISPD) peritonitis guidelines [2], a key for treatment success is related to the stability and compatibility of daptomycin in peritoneal dialysis solutions.

With increasing need to target Gram-positive infections due to resistant organisms (including but not limited to methicillin-resistant *Staphylococcus aureus and* vancomycin-resistant enterococci), the use of daptomycin has been advocated. Daptomycin is a cyclic lipopeptide with bactericidal activity and remains highly active against *S*. *aureus* in stationary growth phase. The drug exhibits superiority in activity against biofilms compared with linezolid and vancomycin [3, 4]. Intraperitoneal route of administration is preferred [5, 6]. Due to its rapid and continuous uptake across the peritoneal membrane into systemic circulation, intraperitoneal administration of daptomycin has also been demonstrated efficacious in treating systemic infection [7]. Previous pharmacokinetic studies [8, 9] suggested the current recommendation of 300 mg daily dose [2]. Alternatively, a loading daptomycin dose of 100 mg/L followed by 20 mg/L maintenance has been used [2, 10]. On the other hand, a higher dosing is recommended for peritonitis from organisms with a high minimum inhibitory concentration (MIC). The potential need of higher daptomycin dose has been suggested by time-kill model and Monte Carlo simulation for resistant organisms such as *Enterococcus faecium* [11]. Study utilizing two-compartmental mathematical pharmacokinetic model [12] suggested that, for methicillin-resistant *Staphylococcus aureus* infection with MIC 0.5 mg/L, a loading dose of 100 mg/L followed by 70 mg/L should be used. Alternatively, an intermittent regimen of 7 mg/kg daily has been proposed [12].

Only a few studies have been performed to assess the stability of daptomycin in peritoneal dialysis, mostly in glucose-based peritoneal dialysis solution [13–16]. Previous studies showed no effect of temperature [15, 16] or glucose concentration of Physioneal [16] and pH-neutral dual-compartment Balance solution [15] on daptomycin stability. Daptomycin was found to be unstable in 5% glucose solution with 15%–20% degradation for 24 h at 25°C [17], and the degree of degradation seems to increase with increasing glucose content (5% and 10%) [16]. While daptomycin was reported to be stable at 25°C for 24 h in low-glucose (1.36% to 2.27%), neutral pH peritoneal dialysis fluid (Physioneal 40, Physioneal 35, or Balance) [15, 16, 18], the stability of daptomycin in other peritoneal dialysis fluids such as UltraBag dextrose 2.5% (pH 5.2) and Stay-Safe Balance (dextrose 2.3%) remains to be determined.

Control of fluid balance during peritoneal dialysis–related peritonitis often requires use of dialysate with dextrose higher than 1.5% or icodextrin. Stability of daptomycin should be tested to facilitate the antibiotic use. There is also uncertainty regarding the duration of daptomycin stability, which can provide the window of opportunity for assistants or nurses to prepare intraperitoneal daptomycin. Accordingly, we sought to examine the stability of daptomycin at a concentration of 70 and 245 μg/mL in three different peritoneal dialysis solutions: glucose-based UltraBag dextrose 2.5% solution, pH-neutral Stay-Safe Balance 2.3%, and UltraBag icodextrin 7.5% solution.

## 2. Materials and Methods

### 2.1. Materials

Daptomycin (AR grade, purity > 98%), purchased from Aladdin Biochemical Technology Co. Ltd. (Shanghai, China), was used as reference standard for the preparation of calibration standards and quantitative analysis of daptomycin in samples. Acetonitrile (high-performance liquid chromatography (HPLC) grade) was purchased from Daejung Chemicals & Metals Co. Ltd. (Korea). Potassium phosphate monobasic (AR grade) was purchased from Meryer (Shanghai, China). Unless specified elsewhere, all reagents were used without further purification. Deionized (DI) water was prepared from Milli-Q water purification system (Millipore, Milford, USA).

Cubicin (daptomycin for injection, 500 mg), manufactured by MSD, was used for the preparation of daptomycin in peritoneal dialysis fluid. Three commercially available peritoneal dialysis fluids (2 L bags), including UltraBag dextrose 2.5% (Baxter Dianeal low-calcium peritoneal dialysis solution with 2.5% dextrose, pH 5.2), UltraBag icodextrin 7.5% (Baxter Extraneal PD solution with 7.5% icodextrin, pH 5.2), and Stay-Safe Balance (Fresenius Stay Safe Balance with 2.3% glucose, pH∼7), were used in this study.

### 2.2. Preparation of Daptomycin Peritoneal Dialysis Fluid

Daptomycin (in Cubicin) was reconstituted with water for injection to obtain 50 mg/mL daptomycin. Appropriate amount of the reconstituted daptomycin was then introduced to the three types of peritoneal dialysis bags, including UltraBag dextrose 2.5%, UltraBag icodextrin 7.5%, and Stay-Safe Balance 2.3%, via the medication port to obtain the final daptomycin concentrations of 70 μg/mL (equivalent to 140 mg/2L, the maintenance level) and 245 μg/mL (equivalent to 490 mg/2L, the loading level). For Stay-Safe Balance, the two compartments were mixed before addition of daptomycin.

### 2.3. Stabilities of Daptomycin in the Prepared Peritoneal Dialysis Bags at Ambient Temperature

To mimic the indoor environment at home/clinical practice, all the peritoneal dialysis bags containing daptomycin were placed at ambient temperature (25°C) during the whole period of stability study. To ensure thorough mixing of daptomycin with the dialysis fluids, the prepared dialysis bags were inverted for at least 10 times upon daptomycin introduction and before sampling.

Samples were collected at 0, 4, 8, 12, 24, and 48 h (after introduction of daptomycin) for UltraBag dextrose 2.5% and UltraBag icodextrin 7.5% and up to 24 h for Stay-Safe Balance 2.3%. The sampling time taken immediately after daptomycin introduction (i.e., hour 0) was considered as baseline. At each sampling time point, an aliquot (5 mL) of peritoneal dialysis fluid was withdrawn from each bag and stored in polypropylene tubes at −20°C until assay (i.e., right after 48 h sampling). Visual inspection on the presence of precipitation and color change was conducted during each sampling.

### 2.4. Sample Analysis by High-Performance Liquid Chromatography With Diode Array Detector (HPLC-DAD)

Samples (20 μL) were introduced into Waters Acquity Ultra-Performance Liquid Chromatography System connected with DAD. Chromatographic separation was achieved via Waters XBridge C_18_ column (4.6 × 250 mm, 5 μm) with the mobile phase containing 0.1 M phosphate buffer (pH 5.5) and acetonitrile (65:35 v/v) at a flow rate of 1 mL/min. The column temperature and the autosampler temperature were set at 25°C and 12°C, respectively. Daptomycin was detected at 223 nm.

Stock daptomycin standard solution (1 mg/mL) was freshly prepared in 50% acetonitrile in DI water. Calibration standards were then prepared by diluting the stock daptomycin standard solution with DI water to final concentrations of 20, 50, 100, 200, 300, and 350 μg/mL daptomycin. Quality control (QC) samples (50 and 300 μg/mL daptomycin) were used for each analytical run. Calibration curve was generated by plotting the daptomycin peak area vs. the concentration of daptomycin in calibration standards. This was then used to determine the concentration of daptomycin in QC and samples.

Prior to sample analysis, the developed method was validated in terms of linearity, accuracy, precision, and stability tests of daptomycin placed in polypropylene tubes under our current sample storage conditions of (i) 24 h in autosampler at 12°C, (ii) 3 h on working bench under ambient, and (iii) four days in freezer at −20°C.

### 2.5. Data Analysis

The concentration of daptomycin obtained from the baseline sample (hour 0) was considered as 100%. The concentrations of daptomycin obtained at other sampling time points were calculated as percentage concentration of daptomycin remaining from the baseline sample. Daptomycin was regarded as stable if it remains at least 90% of the initial concentration. Data were expressed as mean ± SD of three replicates.

## 3. Results

HPLC-DAD analysis was used in this study for the quantification of daptomycin in peritoneal dialysis fluid. The linearity (*r*^2^ > 0.998) is 20 to 350 μg/mL daptomycin, which covers the daptomycin maintenance and loading levels commonly used. The accuracy and precision of two-level QC samples (50 and 300 μg/mL, (*n* = 5 per level) were found to be within 90%–110% (intraday: 95.4–106.0%; interday: 95.3–107.9%) and within 10% RSD (intraday: 4.5%; interday: 6.2%), respectively. Daptomycin samples were found to be stable in autosampler at 12°C for up to 24 h (percentage remaining 98.3 ± 1.9%), on working bench for at least 3 h (percentage remaining 98.8 ± 2.0%), and in freezer under −20°C for up to 4 days (percentage remaining 100.4 ± 3.9%).

**Figure 1:**
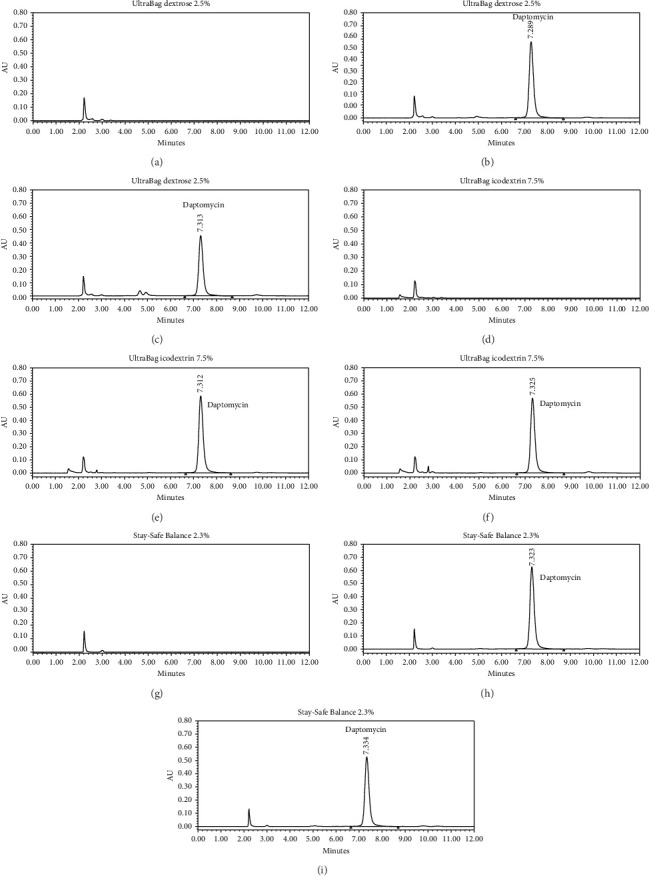
Representative LC chromatograms of blanks (without daptomycin) or prepared samples (240 μg/mL daptomycin) in (a–c) UltraBag dextrose 2.5%, (d–f) UltraBag icodextrin 7.5%, and (g–i) Stay-Safe Balance 2.3% stored at ambient temperature for 0 and 24 h. Daptomycin was eluted at 7.3 min. (a) Blank. (b) Hour 0. (c) Hour 24. (d) Blank. (e) Hour 0. (f) Hour 24. (g) Blank. (h) Hour 0. (i) Hour 24.

As shown in Figure 1, daptomycin in all samples was eluted at 7.3 min without any interference from the studied peritoneal dialysis fluids. Neither precipitation nor change of color was visually observed for all collected samples, indicating physical stability of them over the study period.

The results of stability test of daptomycin in peritoneal dialysis fluids under ambient temperature (25°C) are presented in Table 1. At maintenance level, daptomycin was found to be stable (≥ 90% of initial dose of daptomycin remaining) in UltraBag dextrose 2.5% up to 48 h and up to 24 h in UltraBag icodextrin 7.5% and Stay-Safe Balance 2.3%. At loading level, daptomycin was found to be stable in UltraBag dextrose 2.5% and Stay-Safe Balance 2.3% for 12 h; it was stable for 48 h in UltraBag icodextrin 7.5%.

## 4. Discussion

The present study investigated the stability of daptomycin in glucose-containing peritoneal dialysis fluids and icodextrin-containing peritoneal dialysis fluid. The findings from our study support the daily preparation of intraperitoneal daptomycin, which remained stable in UltraBag dextrose 2.5% (pH 5.2) and Stay-Safe Balance (2.3% glucose, pH∼7) at 25°C for 24 h (and at most 48 h for UltraBag dextrose 2.5%) at its maintenance level (70 μg/mL). These findings were similar to those reports on daptomycin at low/similar level (20 or 50 μg/mL) in low-glucose (1.36%–2.27%), neutral pH (pH 7.4–7.5) dialysis fluid [16, 18]. We confirmed that daptomycin was stable for up to 48 h in UltraBag dextrose 2.5% (pH 5.2).

There are several reasons to investigate the stability of daptomycin. First, antibiotic stability data cannot be extrapolated to newer solution such as icodextrin-based solution. Also, daptomycin was reported to be unstable in high dextrose solutions; neutral pH (pH 6 to 8) buffering fluids with 5% dextrose are recommended to minimize degradation of daptomycin [17]. A limitation of these studies pertains to the deviation from real-world scenario, when many patients develop ultrafiltration problem during acute peritonitis. To mitigate the problem of volume overload and augment the ultrafiltration capacity, patients with peritonitis often require hypertonic solutions (higher dextrose concentration) or icodextrin peritoneal dialysis fluids [19, 20]. Caution should be used in extrapolating results to UltraBag dextrose 2.5% (pH 5.2), UltraBag icodextrin 7.5% (pH 5.2), and Stay-Safe Balance (2.3% glucose, pH∼7). Stability of daptomycin in these peritoneal dialysis solutions—either higher glucose concentration or icodextrin—has to be determined because acute peritoneal inflammation makes patients more vulnerable to reduced ultrafiltration and fluid overload. Data from daptomycin in low-glucose concentration peritoneal dialysis solutions should not be used as a convenient reason to apply to them. As highlighted in the 2022 ISPD peritonitis guidelines for adults [2] and 2024 guidelines for children [21], there have been no data of daptomycin stability in icodextrin-based solutions. The published data on daptomycin in icodextrin were not available until a recent preliminary study on a generic formulation of icodextrin, instead of Extraneal PD solution, in Japan [22]. In addition, the stability of daptomycin at higher dose (> 200 μg/mL) in these fluids has not been explored. Second, stability of daptomycin is crucial for patients who require assisted peritoneal dialysis. The scope of assisted peritoneal dialysis includes those who used to have self-care dialysis but require short-term assistance during acute peritonitis. For patients who lack social support or unable to learn the technique of intraperitoneal daptomycin injection (often owing to limited visual acuity and decreased manual dexterity), they depend on assistants who provide home visit to inject daptomycin. Since the number of visits is limited by the resources, preparation of daptomycin cannot be provided more frequent than daily in most countries.

In comparison with previous trials, our preparation of intraperitoneal daptomycin in two-compartment solution bags is more representative of present-day practice. Ramdas et al. reported that 20 μg/mL daptomycin in neutral medium (pH∼7) of Balance was stable for 3 days when stored at 25°C [15]. In their study, daptomycin was injected into non-glucose compartment of Balance bag (pH 9.2) and stored at 25°C, before mixing with the glucose compartment (pH 3). To reflect usual practice, the two compartments of Stay-Safe Balance 2.3% were mixed (pH∼7) before the introduction of daptomycin in our study.

This trial has direct clinical implication and supports the stability of daptomycin in icodextrin solutions. Measurement of daptomycin in icodextrin by HPLC has previously been reported to be not reproducible, as large variation was observed in replicate samples [16, 18]. Peyro Saint Paul et al. observed lower daptomycin levels in PVC containers compared with glass container, which may reflect the adsorption of daptomycin in container [18]. As shown in Figure 1(d), no interference peak was observed at and around the retention time of daptomycin, indicating that daptomycin is well separated from ingredients in the dialysis fluid. In current study, the recovery of daptomycin collected in 0 h in UltraBag icodextrin 7.5% was 98% (at 70 μg/mL) and ∼90% (at 245 μg/mL), with precision within 5% RSD. We observed that the thorough mixing of daptomycin after introduction into the dialysis bag as well as before sampling is crucial to obtain reproducible results. As shown in Table 1, the stability of daptomycin in UltraBag icodextrin 7.5% at 25°C can be reached for 24 h at maintenance level and 48 h at loading level. Although Suzuki et al. recently reported the stability of daptomycin in generic formulation of icodextrin-based solution at 25°C for 72 h, their findings were restricted to complete light shielding environment, which may overestimate the stability of daptomycin in clinical practice due to its photosensitive and thermal labile properties [22]. Our study, on the other hand, confirmed that daptomycin remained stable for 48 h at loading level under normal indoor environment without light protection.

The stability of daptomycin at maintenance treatment dose is most relevant to patients who require assisted dialysis and procedure of intraperitoneal administration. However, the stability of such dosing in the currently investigated dialysis media has not been reported. Practically, peritoneal dialysis bags are often warmed to 37°C before instillation, with dwell time not exceeding 6 h for daptomycin-containing fluid [16, 18]. Based on our findings, daptomycin loaded at maintenance level (70 μg/mL, equivalent to 140 mg/2L) is stable at 25°C for 24 h in UltraBag icodextrin 7.5%/Stay-Safe Balance 2.3% and up to 48 h in UltraBag dextrose 2.5%. In other words, our findings provide supporting evidence for home visit of nurses every other day to assist patients in drug preparation.

This study has limitations. First, the drug precipitation and color change were assessed by visual examination instead of analytical technique such as light microscopy and UV-Vis spectrophotometry [14]. As a result, subtle color changes or microprecipitation could have been missed. Second, we tested original formulation of daptomycin in the current study. This formulation requires refrigeration for storage, whereas newer lyophilized daptomycin formulations (such as Cubicin RF) can be stored at room temperature as they contain sucrose and sodium hydroxide as the excipient to increase stability and facilitate reconstitution. Although our results may not necessarily be applicable to lyophilized daptomycin [14], newer formulations should be expected to have better stability than the original daptomycin formulation tested.

## 5. Conclusion

We here report the stability of daptomycin in UltraBag dextrose 2.5% (Baxter Dianeal low-calcium peritoneal dialysis solution with 2.5% dextrose, pH 5.2), UltraBag icodextrin 7.5% (Baxter Extraneal PD solution with 7.5% icodextrin, pH 5.2), and Stay-Safe Balance (Fresenius Stay Safe Balance with 2.3% glucose, pH∼7) at 25°C. The data presented here provide a foundational evidence base for nephrologists and nurses to plan the preparation and storage of intraperitoneal daptomycin. The finding of daptomycin stability in icodextrin-based solution is important for patient care in clinical practice.

## Figures and Tables

**Table 1:** Percentage of daptomycin remaining at different time intervals in the three types of peritoneal dialysis bags under ambient condition.

Peritoneal dialysis bag	Time (h)	% of daptomycin remaining (mean ± SD) (*n* = 3)	
		Maintenance level (70 μg/mL)	Loading level (245 μg/mL)
UltraBag dextrose 2.5%	0	100.0	100.0
	4	101.4 ± 1.1	95.8 ± 0.2
	8	97.8 ± 1.7	96.0 ± 0.4
	12	94.9 ± 1.5	95.6 ± 0.5
	24	95.2 ± 0.2	81.6 ± 1.1
	48	90.2 ± 1.0	87.6 ± 0.7

UltraBag icodextrin 7.5%	0	100.0	100.0
	4	94.9 ± 0.8	99.3 ± 0.1
	8	95.4 ± 0.1	99.1 ± 0.2
	12	93.6 ± 1.2	98.3 ± 0.2
	24	94.3 ± 0.6	96.9 ± 0.2
	48	88.9 ± 0.4	95.4 ± 0.2

Stay-Safe Balance dextrose 2.3%	0	100.0	100.0
	4	100.0 ± 0.6	99.6 ± 0.2
	8	100.8 ± 0.6	99.3 ± 0.1
	12	101.4 ± 1.1	97.1 ± 0.5
	24	91.6 ± 0.7	87.2 ± 2.7

## Data Availability

The data that support the findings of this study are available on request from the corresponding authors. The data are not publicly available due to privacy or ethical restrictions.

## References

[1] Cho Y., Chow K. M., Kam-Tao Li P., Runnegar N., Johnson D. W. (2024). Peritoneal Dialysis-Related Infections. *Clinical Journal of the American Society of Nephrology*.

[2] Li P. K., Chow K. M., Cho Y. (2022). ISPD Peritonitis Guideline Recommendations: 2022 Update on Prevention and Treatment. *Peritoneal Dialysis International*.

[3] Kajihara T., Nakamura S., Iwanaga N. (2017). Comparative Efficacies of Daptomycin, Vancomycin, and Linezolid in Experimental Enterococcal Peritonitis. *Journal of Infection and Chemotherapy*.

[4] Zhou Y., Liu M. J., Liao X. Y. (2023). New Attempts to Inhibit Methicillin-Resistant *Staphylococcus aureus* Biofilm? A Combination of Daptomycin and Azithromycin. *Infection and Drug Resistance*.

[5] Pérez Melón C., Borrajo Prol M., Iglesias E., Ferreiro B., Camba Caride M. (2016). Daptomycin in Peritoneal Dialysis, Intraperitoneal or Intravenous. *Nefrologia*.

[6] Bahte S. K., Bertram A., Burkhardt O. (2010). Therapeutic Serum Concentrations of Daptomycin After Intraperitoneal Administration in a Patient With Peritoneal Dialysis-Associated Peritonitis. *Journal of Antimicrobial Chemotherapy*.

[7] Taegtmeyer A. B., Kononowa N., Fasel D., Haschke M., Burkhalter F. (2016). Successful Treatment of a Pacemaker Infection With Intraperitoneal Daptomycin. *Peritoneal Dialysis International*.

[8] Paul L. P. S., Ficheux M., Debruyne D. (2018). Pharmacokinetics of 300 mg/d Intraperitoneal Daptomycin: New Insight from the DaptoDP Study. *Peritoneal Dialysis International*.

[9] Paul L. P. S., Ficheux M., Debruyne D. (2017). Pharmacokinetics of Intraperitoneal Daptomycin in Patients With Peritoneal Dialysis-Related Peritonitis. *Peritoneal Dialysis International*.

[10] Lin S. Y., Ho M. W., Liu J. H. (2011). Successful Salvage of Peritoneal Catheter in Unresolved Methicillin-Resistant staphylococcus Aureus Peritonitis by Combination Treatment with Daptomycin and Rifampin. *Blood Purification*.

[11] Menezes B. K., Alves I. A., Staudt K. J. (2020). Time-kill Curves of Daptomycin and Monte Carlo Simulation for the Treatment of Bacteraemia Caused by Enterococcus Faecium. *Brazilian Journal of Microbiology*.

[12] Charoensareerat T., Taweepunturat T., Rodjun V. (2024). Intraperitoneal Daptomycin Dosing for Peritonitis May Be Inadequate: A Monte Carlo Simulation Approach to Optimize Dosing and Outcomes. *Journal of Chemotherapy*.

[13] Lewis S. J., Alves B., Ratnam S., Churchwell M. D. (2023). Stability and Compatibility of Intraperitoneal Antimicrobials in Peritoneal Dialysate Solutions. *Peritoneal Dialysis International*.

[14] Ling C. W., Sud K., Patel R. (2023). Culture-Directed Antibiotics in Peritoneal Dialysis Solutions: A Systematic Review Focused on Stability and Compatibility. *Journal of Nephrology*.

[15] Ramdas S., Yousaf F., Shastri M. D. (2016). Stability of Daptomycin in Peritoneal Dialysis Solutions Packaged in Dual-Compartment Infusion Bags. *European Journal of Hospital Pharmacy*.

[16] Parra M. A., Campanero M. A., Sadaba B. (2013). Effect of Glucose Concentration on the Stability of Daptomycin in Peritoneal Solutions. *Peritoneal Dialysis International*.

[17] Inman E. L., Kirsch L. E. (1990). Inventor; an Improved Diluent Formulation for Daptomycin.

[18] Paul L. P. S., Albessard F., Gaillard C. (2011). Daptomycin Compatibility in Peritoneal Dialysis Solutions. *Peritoneal Dialysis International*.

[19] Chow K. M., Li P. K., Cho Y. (2023). ISPD Catheter-Related Infection Recommendations: 2023 Update. *Peritoneal Dialysis International*.

[20] Chow K. M., Szeto C. C., Kwan B. C. (2014). Randomized Controlled Study of Icodextrin on the Treatment of Peritoneal Dialysis Patients During Acute Peritonitis. *Nephrology Dialysis Transplantation*.

[21] Warady B. A., Same R., Borzych-Duzalka D. (2024). Clinical Practice Guideline for the Prevention and Management of Peritoneal Dialysis Associated Infections in Children: 2024 Update. *Peritoneal Dialysis International*.

[22] Suzuki H., Kudo K., Uno T. (2024). Assessing the Stability of Daptomycin in Icodextrin-Based Peritoneal Dialysis Solution. *Peritoneal Dialysis International*.

